# In vitro ibuprofen has gene regulatory and anti-inflammatory properties in peripheral blood mononuclear cells of individuals with infection-provoked neurodevelopmental disorders

**DOI:** 10.1007/s10787-026-02282-7

**Published:** 2026-06-11

**Authors:** Velda X. Han, Jessica P. Hayes, Brian Gloss, Ruwani Dissanayake, Xianzhong Lau, Erica Tsang, Hiroya Nishida, Melanie Wong, Shekeeb S. Mohammad, Shrujna Patel, Russell C. Dale

**Affiliations:** 1 Khoo Teck Puat-National University Children’s Medical Institute, National University Health System, Singapore, Singapore; 2 Department of Paediatrics, National University Singapore, Singapore, Singapore; 3 Kids Neuroscience Centre, The Children’s Hospital at Westmead, Faculty of Medicine and Health, University of Sydney, Sydney, NSW Australia; 4 Westmead Research Hub, Westmead Institute for Medical Research, Sydney, NSW, Australia; 5 Australian Genome Research Facility Ltd, Westmead, NSW Australia; 6 Australian Genome Research Facility Ltd, Melbourne, VIC Australia; 7 Department of Brain and Neurosciences, Tokyo Metropolitan Institute of Medical Science, Tokyo, Japan; 8 Department of Allergy and Immunology, The Children’s Hospital at Westmead, Sydney, NSW, Australia; 9 The Children’s Hospital at Westmead Clinical School, Faculty of Medicine and Health, University of Sydney, Sydney, NSW Australia; 10 The Brain and Mind Centre, School of Medical Sciences and Discipline of Child and Adolescent Health, Faculty of Medicine and Health, The University of Sydney, Sydney, NSW Australia

**Keywords:** Neurodevelopmental disorder, Obsessive compulsive disorder, Ibuprofen, Therapeutics, Single-cell RNA seq

## Abstract

Gene-environment interactions, leading to immune-brain cross-talk and neuroinflammation are increasingly recognised in neurodevelopmental disorders (NDDs), such as autistic regression and paediatric acute onset neuropsychiatric syndrome (PANS). Nonsteroidal anti-inflammatory drugs, including ibuprofen, provides relief to some neuropsychiatric symptoms, however, the therapeutic mechanisms are unclear. We describe the benefit of ibuprofen in 18 children (11 males, ages 5–20 years) with infection-provoked deteriorations compatible with PANS (16/18) or autistic regression (7/18), five met criteria for both. Ibuprofen was well tolerated and led to improved emotional control, obsessive–compulsive symptoms, and aggression during infection-related exacerbations or the chronic phase. Single-cell RNA sequencing (scRNA-seq) was performed on peripheral blood mononuclear cells (PBMCs) in two children and their respective age-, sex- matched controls, before and after 24 h of in vitro ibuprofen and/or paracetamol incubation. A total of 45,134 cells were included. At baseline in both cases, there was dysregulation of immune pathways (response to virus, cell killing) and downregulation of ribosomal and translational pathways. Ibuprofen reversed the pro-inflammatory state in both children with reversal of cell killing pathways (granzyme genes) in Case 1, and reversal of viral pathways (interferon genes) in Case 2. Ibuprofen also reversed translation pathways (ribosomal protein genes), and regulation of translation (including *EIF* genes). In vitro paracetamol produced similar but weaker effects than ibuprofen. Children with infection-provoked neurodevelopmental episodes have baseline transcriptional and immune dysregulation in peripheral immune cells. We show that ibuprofen has transcriptional and anti-inflammatory effects, and that scRNA-seq in vitro-based studies can inform disease-modifying therapies for individuals with NDDs.

**Supplementary Information:**

The online version contains supplementary material available at 10.1007/s10787-026-02282-7.

## Introduction

Neurodevelopmental disorders (NDDs) are increasingly recognized to arise from gene-environment interactions, resulting in gene dysregulation and ongoing immune-brain cross-talk (Han et al. 2021). Some children experience neurodevelopmental regression or abrupt-onset neuropsychiatric symptoms such as obsessive–compulsive disorder (OCD) following infections. These clinical syndromes have been termed autistic regression and Paediatric Acute-onset Neuropsychiatric Syndrome (PANS) (Chang et al. 2015; Furley et al. 2023). Multimodal research including neuroimaging, post-mortem brain transcriptomics and cytokine studies supports the presence of neuroinflammation in NDDs (Frick and Pittenger 2016; Vargas et al. 2005; Voineagu et al. 2011).

In support of the neuroimmune hypothesis, nonsteroidal anti-inflammatory drugs (NSAIDs) have been shown to be helpful in open-label studies in children with PANS, and in randomised-controlled trial of psychiatric disorders such as OCD (Brown et al. 2017; Shamabadi et al. 2024; Spartz et al. 2017; Westwell-Roper et al. 2022). It is hypothesised that the anti-inflammatory effects of NSAIDS modulate neuroinflammation, however mechanistic studies are lacking.

In this study, we explored the effects of ibuprofen in children with infection-provoked neurodevelopmental regression. First, we describe our observations of children with NDDs and neuropsychiatric disorders who symptomatically benefited from ibuprofen. Then, we investigated the effects of in vitro ibuprofen on peripheral immune cells from affected children and controls using single-cell RNA sequencing (scRNA-seq).

## Experimental procedures

We first describe clinical observations of 18 children (< 21 years of age) with complex NDDs seen at the Children’s Hospital at Westmead between 2024 and 2025 who had a history of infection-provoked neurodevelopmental deterioration fulfilling criteria for autistic regression (n = 7) and/or PANS (n = 16), who reported clinical benefit with ibuprofen (Chang et al. 2015; Furley et al. 2023). All patients underwent a structured clinical interview using NDD-ECHO (Patel et al. 2025).

### Sample collection

Venous blood samples were collected from two children with infection-provoked neurodevelopmental and neuropsychiatric symptoms (14-year-old male (Case 1) and 12-year-old female (Case 2)) and two age- and sex-matched healthy controls for in vitro ibuprofen and paracetamol testing. Samples were collected in Vacutainer ACD tubes (BD Biosciences, Cat. No. BD367756). Peripheral blood mononuclear cells (PBMCs) were isolated and stored as previously described (Han et al. 2025b).

Samples were taken from patients in the chronic phase of their disease, 11 years (Case 1) and 9 years (Case 2) after their first episode, not during an exacerbation, and none of the patients or controls had significant infections or allergies one month prior to blood taking. The two patients were not taking antibiotics or immunotherapy at the time of sampling. The controls did not have NDDs, autoimmune conditions or significant allergies.

### In vitro ibuprofen and paracetamol treatment of PBMCs for single-cell RNA sequencing

Frozen PBMC aliquots from two patients and two controls were thawed rapidly in a 37 °C water bath, followed by washing with thawing medium consisting of RPMI-1640 supplemented with 10% fetal bovine serum (FBS), 1% GlutaMAX™, and 1% HEPES. The cells were then incubated in culture medium (RPMI 1640 supplemented with 10% FBS and 1% GlutaMAX™) at 37 °C in a 5% CO_2_ atmosphere for 3 h. Following the incubation, the cells were treated with 40 µg/mL ibuprofen, 8 µg/mL paracetamol or media (0.08% ethanol) in culture media for 24 h at 37 °C in a 5% CO_2_ atmosphere. Concentrations of ibuprofen and paracetamol were determined by maximum recommended plasma concentrations reported by Therapeutic Goods Administration (https://www.tga.gov.au/resources/auspar/auspar-ibuprofen-0) and independent in-human studies (García Aguirre et al. 2019; Legg et al. 2014; Portolés et al. 2003; Trocóniz et al. 2000; Yin et al. 2001).

### Single-cell RNA sequencing of PBMCs

Following 24-h incubation with ibuprofen, paracetamol or media, the cells (2 patient-media, 2 control-media, 2 patient-ibuprofen, 1 patient-paracetamol, 1 control-ibuprofen sample) were harvested, washed, and stained with DAPI (1:100). Live (DAPI‑negative) cells were sorted using the BD FACSAria™ III Cell Sorter in accordance with the manufacturer’s protocol at the Westmead Cytometry Core Facility, Westmead Research Hub (Westmead, Australia), and subsequently loaded into HIVE™ devices (Honeycomb Biotechnologies, Inc.). HIVE™ devices were loaded with approximately 30,000 cells from each sample in 1 mL of DPBS + 1% FBS followed by 3 mL of cell media (DPBS + 1% FBS). The scRNA-seq workflow is described in Supplementary Methods.

### Bioinformatic analysis

ScRNA-seq data were analysed using the *Seurat* package, analysis workflow in Supplementary Methods. Pathway enrichment analysis (FDR < 0.05) was performed via Over Representation Analysis Gene Ontology (ORA GO) using the *clusterProfiler* package. Bar, dot, violin plots, and heatmaps were plotted using *ggplot2* package.

### Ethics approval

Ethical approval was granted by Sydney Children’s Hospitals Network Human Research Ethics Committee (HREC/18/SCHN/227, 2021/ETH00356), and written consent obtained from families.

## Results

### Clinical data

We describe 18 children (11 males, 7 females) aged 5–20 years with a history of infection-provoked NDDs. 16/18 met criteria for PANS, and 7/18 had a history of autistic regression (5 had both features). The follow-up was median 6 years (range 1.5–18 years) since first neurodevelopmental concerns. DSM-V diagnoses included ASD (12/18), OCD (16/18), attention-deficit/hyperactivity disorder (ADHD; 10/18), and anxiety disorder (9/18), with additional features such as rage, self-injurious behaviour or aggression (9/18) and tics (5/18) (Table 1).

**Table 1:** Clinical characteristics, ibuprofen use and other conventional therapies in children with infection-provoked neurodevelopmental disorders

Case	Age first symptoms	Age	Sex	Conventional NDD and psychiatric diagnosis	PANS criteria	Autistic regression	Use of Ibuprofen	Symptoms improved by Ibuprofen	Duration of Ibuprofen	Ibuprofen repeated	Conventional therapies	Other immune treatment
1	3	14	M	ASD, ADHD, Tourette, OCD, anxiety, rage	+		Symptomatic	OCD, anxiety, rage	PRN	Yes	Aripiprazole, Methylphenidate, Fluoxetine, Clonidine, CBD	Azithromycin
2	3	12	F	ASD, OCD, anxiety	+	+	Infection provoked regression	Emotional control	3 days	Yes	–	–
3	1.5	6	M	ASD, ADHD, OCD, tics, rage	+	+	Infection provoked regression	Emotional control, tics, speech, attention, aggression	Ongoing	Ongoing	CBD	IVIG
4	1.1	6	M	ASD, OCD, anxiety	+	+	Infection provoked regression	Anxiety, rigidity, aggression	3 days	Yes	CBD	Azithromycin
5	1.5	7	M	ASD, SIB, aggression		+	Infection provoked regression	Aggression, self-injury	2 days	Yes	Risperidone, Clonidine, Valproate	Azithromycin
6	5	9	F	ASD, ADHD, OCD	+	+	Infection provoked regression	Speech, toileting, motor skills, tics, attention	5 days	Yes	–	–
7	3.5	5	M	OCD, eating restriction	+		Infection provoked flares	OCD, eating restriction, repetitive behaviour	2 days	Yes	–	–
8	5	7	F	OCD, ADHD, anxiety, ODD	+		Infection provoked flares	OCD, anxiety	2 days	Yes	Guanfacine	Azithromycin
9	2	8	M	ASD, ADHD, OCD, aggression	+	+	Infection provoked flares	Emotional control, attention, speech, eye contact	2 days	Yes	Aripiprazole, Clonidine, Fluoxetine	Augmentin
10	2	8	F	ASD, ADHD, OCD, anxiety, rage	+		Infection provoked flares	OCD, rage, sensory, anxiety	2 days	Yes	CBD	Azithromycin
11	5.5	8	F	ADHD, OCD, anxiety, separation anxiety, ODD	+		Infection associated flares	OCD, separation anxiety, anxiety	3 days	Yes	Aripiprazole, Fluoxetine	IVIG, Azithromcyin
12	2	9	M	ASD, ADHD, OCD, rage	+		Infection provoked flares	OCD, anxiety	2 days	Yes	Lysdexamphetamine	IVIG, Azithromcyin
13	2	9	M	ASD, ADHD, anxiety	+		Infection provoked flares	Hallucinations, fight and flight response	5 days	No	Methylphenidate	
14	3	9	F	OCD, tics anxiety, rage	+		Infection provoked flares	Anxiety, OCD, tics, urinary wetness, appetite, sleep	3 days	Yes	–	Azithromycin
15	2	12	M	ASD, Tourette, OCD	+		Infection provoked flares	Tics, emotional control	3 days	Yes	Aripiprazole, Clonidine, Fluoxetine	Azithromycin
16	6	13	M	ADHD, OCD, rage	+		Infection provoked flares	Emotional control, OCD, aggression, sleep	5 days	Yes	Fluvoxamine	Azithromycin, prednisolone
17	5	13	F	OCD, Tourette	+		Infection provoked flares	Emotional control, OCD, tics	3 days	No	Brexpiprazole, Lamotrigine, Clonidine	IVIG, Azithromcyin
18	1.5	20	M	ASD, ID, OCD, anxiety, rage		+	Symptomatic	Rage, irritability, aggression	PRN	Yes	Aripiprazole, Guanfacine	

The age of first symptoms is the age of first developmental concerns according to family

ID, intellectual disability; ASD, autistic spectrum disorder; ADHD, attention deficit hyperactivity disorder; CBD, cannabidiol; IVIG, intravenous immunoglobulin; OCD, obsessive compulsive disorder; ODD, oppositional defiant disorder; SIB, self-injurious behaviour; PRN, as needed

The children or family members observed clinical benefit of ibuprofen in the context of infection-provoked regression episodes (5/18), infection-provoked neuropsychiatric flares (11/18), or during the chronic phase for symptomatic treatment of behaviour, as required (2/18), with duration of ibuprofen ranging from 2 days to ongoing treatment (median 3 days) (Table 1). Symptomatic improvement was observed in emotional control or anxiety (13/18), OCD symptoms (9/18), aggression or rage (6/18), and less commonly for tics, speech, attention and sleep (Table 1). Ibuprofen was repeatedly used in most patients (16/18) during infection-provoked episodes, and families reported generally consistent and useful effects. No patients experienced significant side effects. Concomitant medications and other immune therapies were also used (Table 1).

### scRNA-seq of PBMC of patient-media vs control-media, and in vitro treatment with ibuprofen

A total of 45,134 cells were included in the PBMC samples before and after in vitro ibuprofen in Case 1, Case 2 and two controls (each matched to one Case). UMAP shows 5 main cell types: CD8 T cells, CD4 T cells, B cells, monocytes and NK cells (Supplementary Fig. 1 for Case 1, and Supplementary Fig. 2 for Case 2). Across cell types, the number of differentially expressed genes (DEGs) in the patient-media versus control-media comparison ranged from 43 to 635 (Case 1), 41 to 608 (Case 2), and the DEGs in patient-ibuprofen versus patient-media comparison ranged from 30 to 794 (Case 1) and 55 to 3325 (Case 2), respectively.

**Fig. 1:**
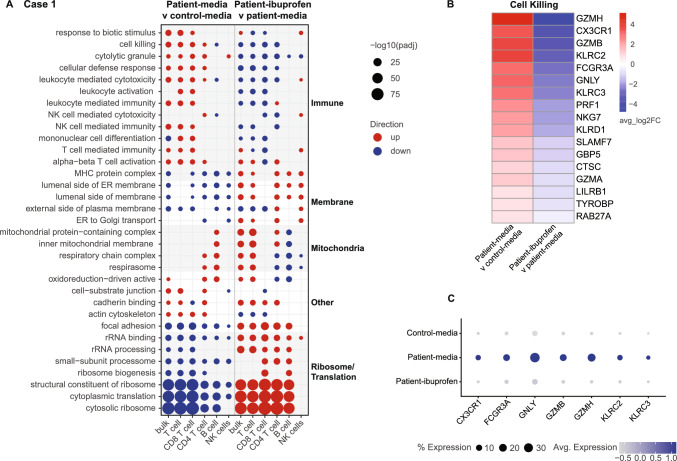
scRNA-seq pathway enrichment and representative genes in Case 1 PBMCs with in vitro ibuprofen treatment. **A** Top 5 upregulated (red) and downregulated (blue) pathways in bulk PBMCs, CD8 T cells, CD4 T cells, B cells, and NK cells across comparisons. Dot size represents –log10 (padj), where padj is adjusted p value. In patient-media versus control-media, broad immune pathways (response to biotic stimulus, cell killing, cellular defense response) were upregulated, whereas ribosomal pathways (cytosolic ribosome, cytoplasmic translation) were downregulated. These changes were reversed in the patient-ibuprofen versus patient-media comparison. Pathways involving 5 or more cell types across comparisons were included in this dotplot. **B** Heat map of the ‘cell killing’ pathway showing 17 genes upregulated in patient-media versus control-media comparison, which were then downregulated in patient-ibuprofen versus patient-media comparison. Average log2 fold change (log2FC) for each gene across conditions. **C** Dot plots of representative genes (*CX3CR1, FCGR3A, GNLY, GZMB, GZMH, KLRC2, KLRC3*) showing average gene expression upregulated in patient-media cells compared to control-media cells and downregulated with in vitro ibuprofen treatment. Dot size represents percentage of cells expressing the gene

**Fig. 2:**
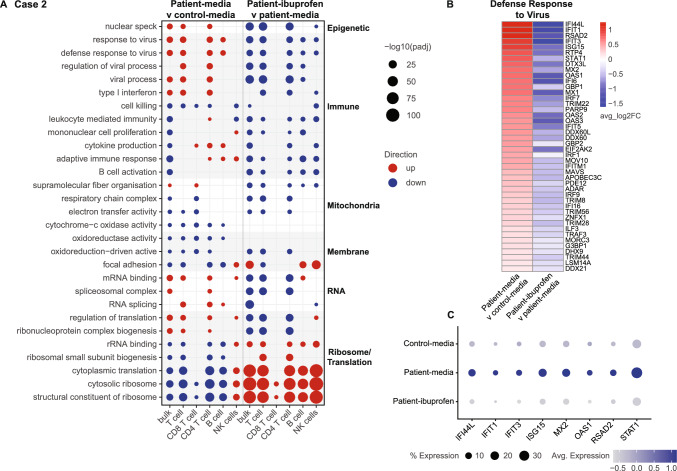
scRNA-seq pathway enrichment and representative genes in Case 2 with in vitro ibuprofen treatment. **A** Top 5 upregulated (red) and downregulated (blue) pathways in bulk PBMCs, CD8 T cells, CD4 T cells, B cells, and NK cells. Dot size represents –log10 (padj), where padj is adjusted p value. In patient-media versus control-media, immune pathways (response to virus, defense response to virus, viral process) were upregulated, while adaptive immune, B cell activation, and ribosomal pathways were downregulated. These changes were generally reversed with in vitro ibuprofen treatment. Pathways involving 5 or more cell types across comparisons were included in this dotplot. **B** Heat map of the ‘defense response to virus’ pathway showing 44 genes upregulated in patient-media versus control-media comparison, and downregulated with in vitro ibuprofen treatment. Displays average log2FC for each gene across conditions. **C** Dot plots show representative genes (*IFI44L, IFIT1, IFIT3, ISG15, MX2, OAS1, RSAD2, STAT1*) illustrating average gene expression upregulated in patient-media cells compared to control-media cells, and downregulated with in vitro ibuprofen treatment. Dot size represents percentage of cells expressing the gene

### Case 1 history

This boy developed repetitive speech and sensory interests and was diagnosed with ASD at age 3. At age 7, he had fever, sore throat, and abrupt-onset OCD characterised by checking, fear of safety, hoarding, and onset of tics. He hoarded plastic bags and shopping receipts for hours each day. After two years of OCD refractory to psychology and fluoxetine, a trial of azithromycin produced a near complete remission of his OCD (CYBOCS from 25/40 to 6/40). Since the first event, he has baseline ASD symptoms, tics and ADHD, but achieved mainstream education, with good social intent and use of language. However, approximately 2–4 times per year he has dramatic exacerbations, triggered by sore throats, mouth ulcers or other infections, with abrupt-onset OCD, hallucinations, impulsivity, dilated pupils, agitation, fight and flight responses, and negative thoughts of death. These infection-associated exacerbations may last a month, and family observe that ibuprofen significantly improves his OCD and emotional control for 2–6 h. He requires multiple conventional medications to control his complex symptoms (Table 1). Neuroimaging and trio exome sequencing was negative.

### scRNA-seq in vitro ibuprofen therapeutics

The top 5 upregulated and downregulated pathways in bulk PBMCs, CD8 T cells, CD4 T cells, B cells, and NK cells in Case 1 are shown at baseline (patient-media v control-media) and after in vitro ibuprofen treatment (patient-ibuprofen v patient-media). In patient-media versus control-media comparison, broad immune-related (response to biotic stimulus, cell killing, cellular defense response), mitochondrial pathways were upregulated, whereas cell membrane, and ribosomal pathways (cytosolic ribosome, cytoplasmic translation), were downregulated (Fig. 1A). These pathways were reversed in the patient-ibuprofen versus patient-media comparison.

We profiled the ‘cell killing’ pathway using bulk analysis of scRNA-seq data and identified 17 genes that were significantly upregulated in patient-media versus control-media comparison and also significantly downregulated in patient-ibuprofen versus patient-media comparison (Fig. 1B). These genes were enriched for immune signalling (chemokine receptors: *CX3CR1,* signaling adaptors*: FCGR3A*), cytotoxic effectors (granule proteins: granzyme *GZM* genes, granule trafficking), and NK cell receptors (activating receptors: *KLRC2, KLRC3,* inhibitory receptors) (Table 2). There was reduction of gene expression in patient cells treated with ibuprofen towards control levels (Fig. 1C).

**Table 2:** ‘Cell killing’ pathway: 17 genes within this pathway were upregulated in Case 1 patient-media versus control-media comparison and downregulated in patient-ibuprofen versus patient-media comparison

Main category	Subcategory	Genes
Immune signaling and migration	Chemokine and migration receptors	*CX3CR1*
	Signaling adaptor/regulators	*FCGR3A, TYROBP*
	Inflammation and immune effector proteins	*GBP5*
Cytotoxic effectors	Granule proteins	*GNLY, GZMB, GZMH, GZMA, PRF1, NKG7*
	Granule trafficking and activation	*RAB27A, CTSC*
NK cell receptors and regulators	Activating receptors	*KLRC2, KLRC3, SLAMF7*
	Inhibitory receptors	*KLRD1, LILRB1*

These genes are categorized into main functions: immune signaling & migration, cytotoxic effectors, NK cell receptors & regulators

### Case 2 history

Up to the age of 3, this girl’s parents described her development to be ahead of her two neurotypical older brothers. Aged 3, she developed a dry cough with sore throat, fever and malaise, attributed to a viral infection and no antibiotics were prescribed. The next week, there was a drastic loss of speech resulting in mutism, new-onset anxiety and disruptions in eating and sleep patterns. She became completely silent, and developed significant separation anxiety. By 3.3 years, she lost eye contact gradually over the next 6 months. There was significant reduction in imaginative play, replaced by restricted play “holding on to toy with flapping”. She developed obsessive–compulsive behaviours including eating dirt, visual stimming, watching water flow from the tap, and repetitive movements of crossing legs with grunting sounds attributed to stereotypic behaviour. At age 12 years, she remains non-verbal, has elevated anxiety, social difficulties and intellectual disability with cognitive function equivalent to a 2-year-old. Conventional medications apart from risperidone have not helped. MRI brain, CSF, EEG, and trio exome sequencing were normal. The family consistently observes that during periods of heightened anxiety, distress, 2–3 days of ibuprofen will ‘calm her’ towards her baseline, and is used every other month for this purpose.

### scRNA-seq in vitro ibuprofen therapeutics

The top 5 upregulated and downregulated pathways in bulk PBMCs, CD8 T cells, CD4 T cells, B cells, and NK cells in Case 2 are shown in Fig. 2. In patient-media versus control-media comparison, epigenetic (nuclear speck), immune-related (response to virus, defense response to virus, viral process), RNA-related pathways (mRNA binding, spliceosomal complex) were upregulated, while other immune pathways (adaptive immune response, B cell activation, and cell killing), mitochondrial, cell membrane, and ribosomal pathways (structural constituent of ribosome, cytosolic ribosome, and cytoplasmic translation) were downregulated (Fig. 2A). These changes were generally reversed in the patient-ibuprofen versus patient-media comparison, except for mitochondrial and cell membrane pathways.

We profiled the ‘defense response to virus’ pathway from bulk analysis of scRNA-seq and identified 44 genes that were upregulated in patient-media versus control-media and subsequently downregulated in patient-ibuprofen versus patient-media (Fig. 2B). These genes were enriched for interferon signalling, RNA metabolism/sensing, and stress and translational control (Table 3). There was reduction of gene expression in patient cells treated with ibuprofen towards control levels (Fig. 2C).

**Table 3:** ‘Defense response to virus’ pathway: 44 genes within this pathway were upregulated in Case 2 patient-media versus control-media comparison and downregulated in patient-ibuprofen versus patient-media comparison

Main category	Subcategory	Genes
Interferon signaling and regulation	Viral RNA/DNA recognition & signaling	*ADAR, MAVS, IFI16*
	Transcription factors and signal transducers	*IRF1, IRF7, IRF9, STAT1, TRAF3*
	Interferon-induced effectors/ISGs	*ISG15, RSAD2, MX1, MX2, IFIT1, IFIT3, IFIT5, IFI6, IFI44L, IFITM1, OAS1, OAS2, OAS3, GBP1, GBP2*
	Interferon Response Modulators	*RTP4, TRIM8, TRIM22, TRIM28, TRIM44, TRIM56, PARP9, DTX3L*
RNA metabolism and sensing	RNA helicases and sensors	*DHX9, DDX21, DDX60, DDX60L, MOV10, ZNFX1, LSM14A, G3BP1*
	RNA editing/modification	*APOBEC3C, PDE12, ILF3*
Stress and translational control	Stress response and translation inhibition	*EIF2AK2, MORC3*

These genes are categorized into main functions: interferon signaling & regulation, RNA metabolism & sensing, stress & translational control

**Fig. 3:**
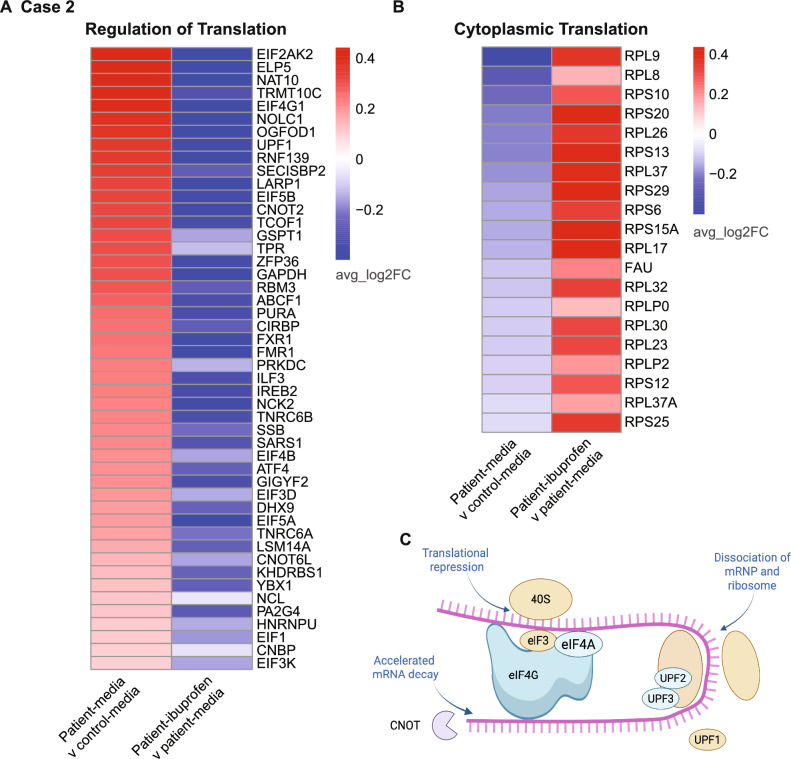
*‘*Regulation of translation’ and ‘cytoplasmic translation’ pathways. **A** Heat map of the ‘regulation of translation’ pathway showing 48 genes upregulated in patient-media versus control-media comparison, which were downregulated with in vitro ibuprofen treatment. Displays average log2FC for each gene across conditions. **B** Heat map of the ‘cytoplasmic translation’ pathway showing 20 genes downregulated in patient-media versus control-media and upregulated with in vitro ibuprofen treatment. Displays average log2FC for each gene across conditions. **C** Graphic depicting the ‘Nonsense mediated decay’ (NMD) pathway. The NMD pathway regulates translation by degrading faulty mRNAs, especially under environmental stress like infection or oxidative damage. NMD acts as both a quality control mechanism and a regulator of gene expression and translation. The 40S ribosomal subunit is a small component composed of ribosomal proteins S, which play a crucial role in the process of translation. Factors such as the *EIF* family reduce translation, while *UPF1, CNOT2,* and *CNOT6L* promote decay of defective mRNAs

In the upregulated ‘regulation of translation’ pathway from bulk analysis of scRNA-seq analysis (Fig. 3A, Table 4), 48 genes were upregulated in patient-media versus control-media and subsequently downregulated in patient-ibuprofen versus patient-media. These genes were enriched for pathways related to translational control (including *EIF* genes), RNA processing and stress response (Table 4).

**Table 4:** ‘Regulation of translation’ pathway: 48 genes within this pathway were upregulated in Case 2 patient-media versus control-media comparison and downregulated in patient-ibuprofen versus patient-media comparison

Category	Subcategory	Genes
Translational control	Initiation/elongation factors	*EIF2AK2, EIF4G1, EIF4B, EIF3D, EIF3K EIF5B, EIF1, GSPT1, EIF5A*
	Translational regulators	*LARP1, YBX1, ABCF1, PA2G4, CNBP, GIGYF2, ATF4, ILF3*
Disruption of messenger ribonucleoprotein (mRNP) complexes	mRNP assembly/remodeling	*HNRNPU, KHDRBS1, CIRBP, FXR1, FMR1, RBM3, LSM14A, NCK2*
	Nuclear export/structural factors	*NCL, TPR, ELP5, NAT10, NOLC1*
Accelerated decay of abnormal transcripts	Core decay/surveillance	*UPF1, TNRC6A, TNRC6B, ZFP36, CNOT2, CNOT6L*
	RNA stability/decay-linked	*SSB, IREB2, RNF139, OGFOD1, SECISBP2*
Removal of incomplete/stress-related polypeptides	Stress-response/proteostasis	*PRKDC, PURA, GAPDH*
	Ribosome/translation/mitochondrial QC	*DHX9, SARS1, TCOF1, TRMT10C*

These genes are categorized into main functions: translational control, disruption of messenger ribonucleoprotein (mRNP) complexes, accelerated decay of abnormal transcripts, removal of incomplete/stress-related polypeptides

By contrast, the ‘cytoplasmic translation’ pathway from bulk scRNA-seq analysis (Fig. 3B, Table 5), 20 genes were downregulated in patient-media versus control-media and subsequently upregulated in patient-ibuprofen versus patient-media. These genes were enriched for cytoplasmic ribosomal proteins, including both small (*RPS*) and large (*RPL*) subunit components.

**Table 5:** ‘Cytoplasmic translation’ pathway: 20 genes within this pathway were downregulated in Case 2 patient-media versus control-media and upregulated in patient-ibuprofen versus patient-media comparison

Category	Subcategory	Genes
Ribosome/translation	Cytoplasmic ribosomal proteins	*RPS25, RPS12, RPS15A, RPS6, RPS29, RPS13, RPS20, RPS10*
	Cytoplasmic ribosomal proteins (large subunit)	*RPL37A, RPLP2, RPL23, RPL30, RPLP0, RPL32, FAU, RPL17, RPL37, RPL26, RPL8, RPL9*

These genes had main functions in ribosome/translation

### Effect of paracetamol on patient PBMCs

We examined the effects of in vitro paracetamol treatment on PBMCs in Case 1. Similar to ibuprofen, the upregulated immune pathways (regulation of response to biotic stimulus, cell killing) at baseline (patient-media) were downregulated by paracetamol, and the downregulated ribosomal pathways at baseline were upregulated by paracetamol (Supplementary Fig. 3). However at the examined physiological doses, ibuprofen upregulated ribosomal pathways (cytosolic ribosome, structural constituent of ribosome) and downregulated immune pathways (defense response to virus, type 1 interferon production) to a greater degree than paracetamol (Supplementary Fig. 4).

### Effect of ibuprofen on control cells

In vitro ibuprofen treatment in control cells showed upregulated immune pathway activity and downregulated ribosomal and translational processes, highlighting a difference from its effects on patient-specific cells (Supplementary Fig. 5).

## Discussion

A significant proportion of individuals with complex NDDs are refractory to conventional treatments. Most treatments only offer symptom relief, with few disease-modifying options available. Some children suffer infection-provoked neurodevelopmental or neuropsychiatric episodes, described as the clinical syndromes PANS and autistic regression (Boterberg et al. 2019; Chang et al. 2015; Stefanatos 2008). We previously proposed that these clinical syndromes are related to gene-environment interactions (gene regulation and epigenetic) affecting the immune system and brain, with ongoing environmental factors triggering immune-brain cross-talk (Han et al. 2025a). Families observed that anti-inflammatory approaches such as ibuprofen can result in symptomatic benefit, particularly in emotional control, which is often used for short periods during these infection provoked exacerbations (Brown et al. 2017; Shamabadi et al. 2024; Spartz et al. 2017; Westwell-Roper et al. 2022).

We explored the baseline gene regulation in two children, and effects of ibuprofen using scRNA-seq of PBMCs. We showed dysregulation of broad immune pathways (e.g. response to virus, cell killing) and downregulation of ribosomal and translational pathways at baseline, which were reversed by ibuprofen treatment. Although immune pathways were generally upregulated in these two cases, some immune pathways were downregulated, suggesting ‘dysregulation’ is a better term. Patients displayed similar but distinct immune signatures: Case 1 had a “bacterial” profile driven by granzymes, while Case 2 showed a “viral” profile driven by interferons. We propose that PANS involves shared immune dysregulation, though the specific pathways may vary among individuals. Unlike our prior studies relying on bulk or cohort scRNAseq (Han et al. 2025a, 2025b; Hayes et al. 2026), this report demonstrates that individual-level scRNAseq reveals unique immune pathway differences. However, these findings should be considered exploratory in view of the small sample size. In vitro paracetamol produced similar but weaker effects (at the chosen physiological concentrations) than ibuprofen. Paracetamol has anti-inflammatory effects, weaker than NSAIDs, acting as a weakly selective COX2 inhibitor via peroxidase activity inhibition of COX1 and COX2 (Graham et al. 2013). Similar gene expression changes were observed in both paracetamol- and ibuprofen-treated cells, suggest both drugs share anti-inflammatory or immunomodulatory actions, however other cellular effects within this in vitro cellular model are possible.

In our study, we identified that ibuprofen has substantial effects on gene regulation. The chemical name of ibuprofen is Iso-butylphenyl-propionic acid, and proprionic acid is a known epigenetic metabolite produced by bacteria in the large bowel which can modulate neuroinflammation (Duscha et al. 2020). Extensive research shows that ibuprofen affects cancer biology by altering histone acetylation and methylation (Shen et al. 2020) and reduces the gene expression of HDACs and KDM6A/B, modifying histone marks in cancer cells (Shen et al. 2020).

The findings in Case 2 revealed gene expression control at the RNA-ribosome interface. The coordinated upregulation of translation-regulatory pathways and downregulation of cytoplasmic translation suggest activation of nonsense-mediated mRNA decay (NMD). NMD, acts as a quality-control system to block translation of defective transcripts (Fig. 3C). Environmental stress disrupts mRNA translation and activates NMD and RNA surveillance pathways, reducing overall protein synthesis and shifting resources toward stress responses (Lykke-Andersen and Jensen 2015). Translation is essential for neurodevelopment, thus disruptions in these processes can impact neuronal growth, differentiation, and synaptic function, with dysfunction linked to disorders like ASD (Chen et al. 2019). We showed that this RNA–protein interface is modified by ibuprofen, at least transiently.

This study’s strength lies in examining cell-type–specific changes by testing ibuprofen and paracetamol at physiological concentrations in children with a history of infection-triggered neurodevelopmental regression. Limitations include a small sample size that limits generalizability, possible effects of psychotropic medications on gene expression in Case 1, and potential reporting bias of our clinical observations due to open-label methodology. Larger cohorts using individual-level blood scRNAseq in PANS are needed to determine whether patient subtypes exist or if pathway variations correlate with symptoms and disease phases. Additionally, future studies incorporating in vitro dose–response analyses, and time-course experiments will help further distinguish drug-specific effects. Lastly, our analyses focused only on peripheral immune cells, while brain effects can only be explored in animal models.

Despite these limitations, our findings provide preliminary evidence that ibuprofen modulates dysregulated gene expression in children with neuropsychiatric relapses. Large randomized controlled trials are needed to determine clinical efficacy of ibuprofen or related molecules. Multi-omics approaches, such as epigenetics, proteomics, and targeted immune studies of T cell subsets (Treg, T gamma delta, Th17), may reveal diagnostic and therapeutic biomarkers for children with NDDs.

## Supplementary Information

Below is the link to the electronic supplementary material.

Supplementary file 1

Supplementary file 2

## Data Availability

Anonymised data not published within this article will be made available by request from any qualified investigator.
