# Supplementary material for: In vitro ibuprofen has gene regulatory and anti-inflammatory properties in peripheral blood mononuclear cells of individuals with infection-provoked neurodevelopmental disorders
Source: Inflammopharmacology. 2026 Jun 11;34(7):4951–62. doi: 10.1007/s10787-026-02282-7 (PMC13391669; doi:10.1007/s10787-026-02282-7)
Supplement: Supplementary file 1 [file 10787_2026_2282_MOESM1_ESM.pdf]

Supplementary Figure 1: Case 1 scRNAseq, UMAP

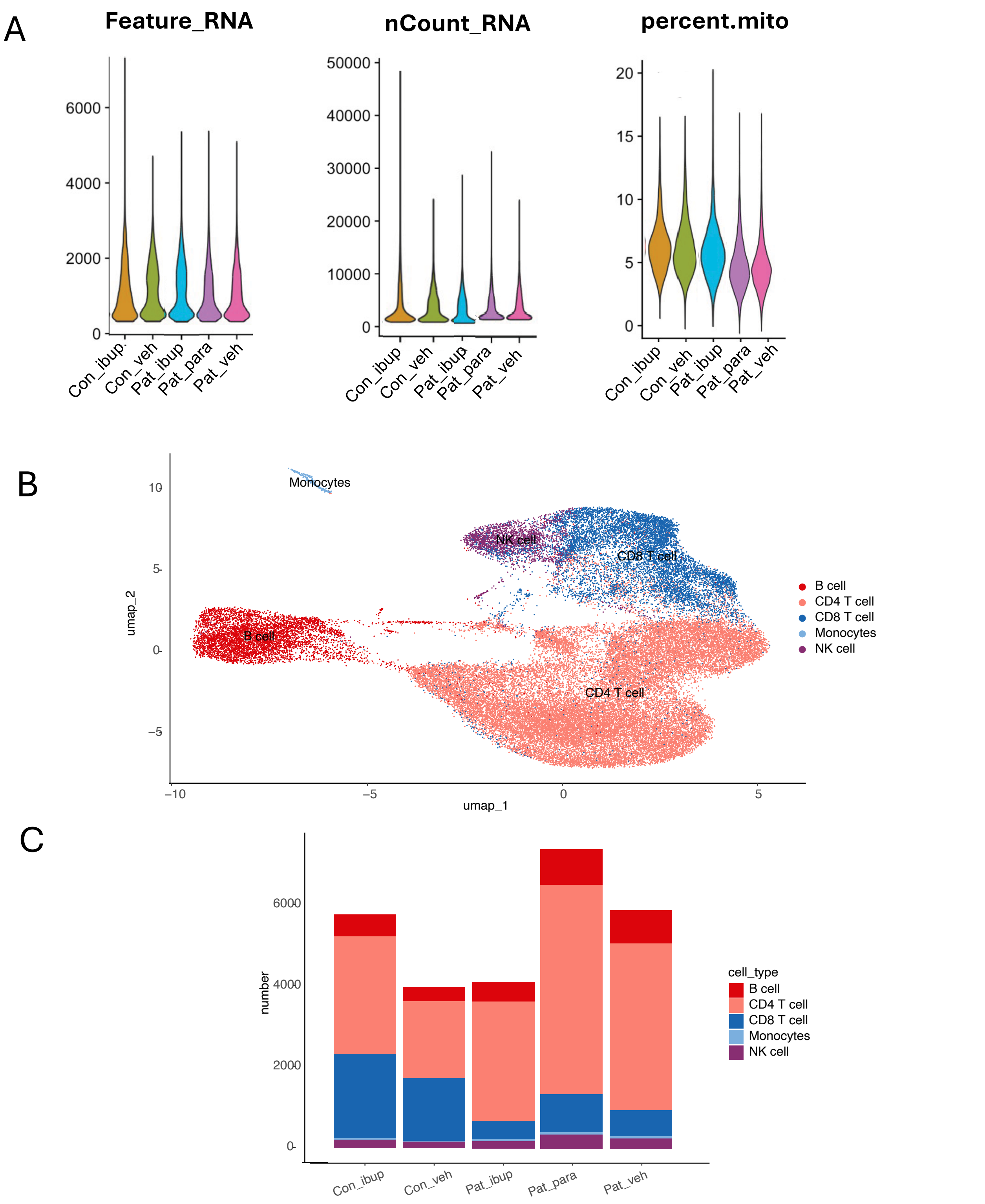

A) Quality control metrics of scRNA-seq including nFeature, nCount, and percent mitochondrial genes expression
B) Uniform Manifold Approximation and Projection (UMAP) visualization showing five cell types: B cells, CD4 T cells, CD8 T cells, monocytes, and NK cells
C) Distribution of cell numbers across samples.

Supplementary Figure 2: Case 2 scRNAseq, UMAP

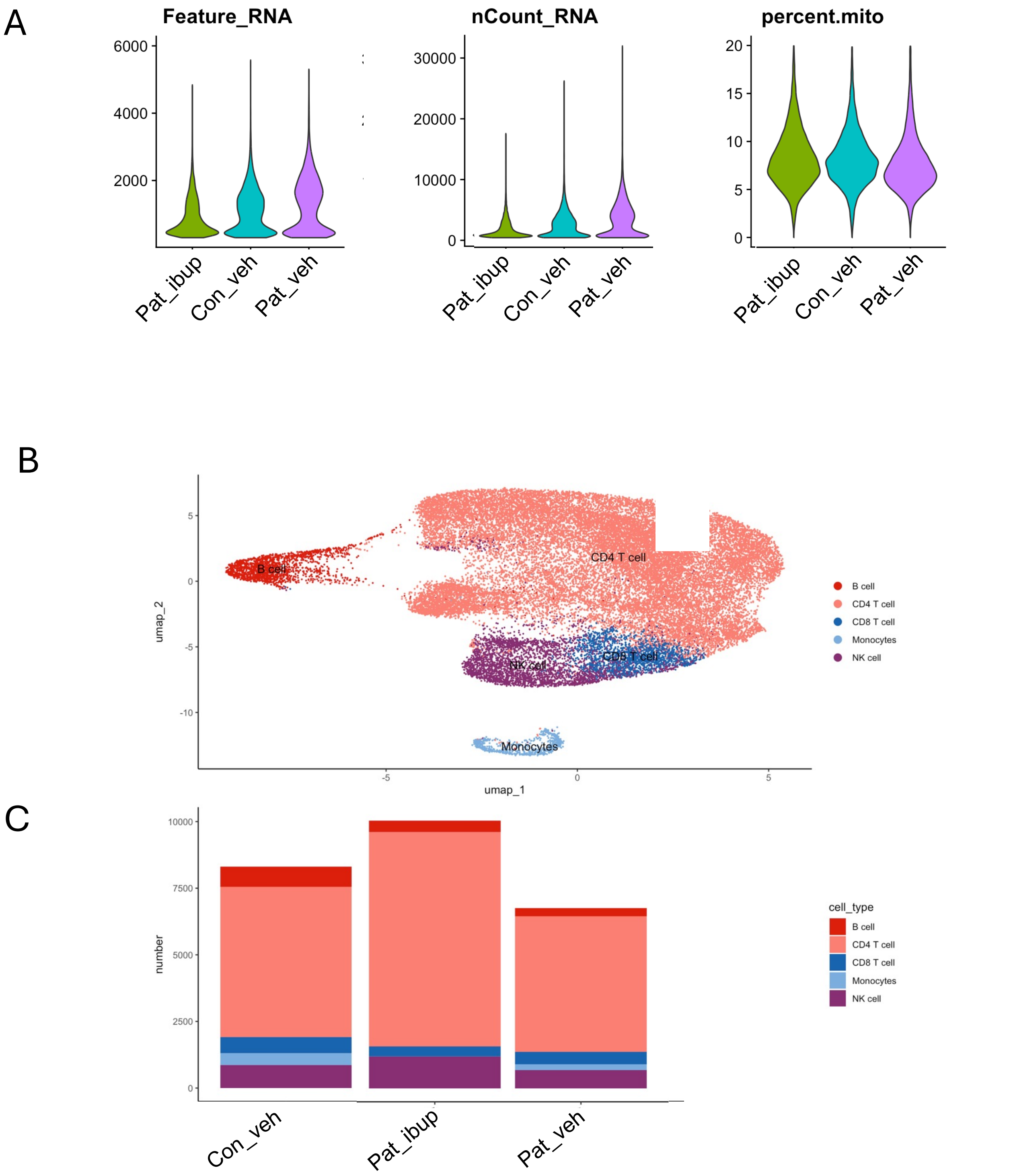

A) Quality control metrics of scRNA-seq including nFeature, nCount, and percent mitochondrial genes expression
B) Uniform Manifold Approximation and Projection (UMAP) visualization showing five cell types: B cells, CD4 T cells, CD8 T cells, monocytes, and NK cells
C) Distribution of cell numbers across samples.

Supplementary Figure 3: Patient-paracetamol versus patient-media dotplot

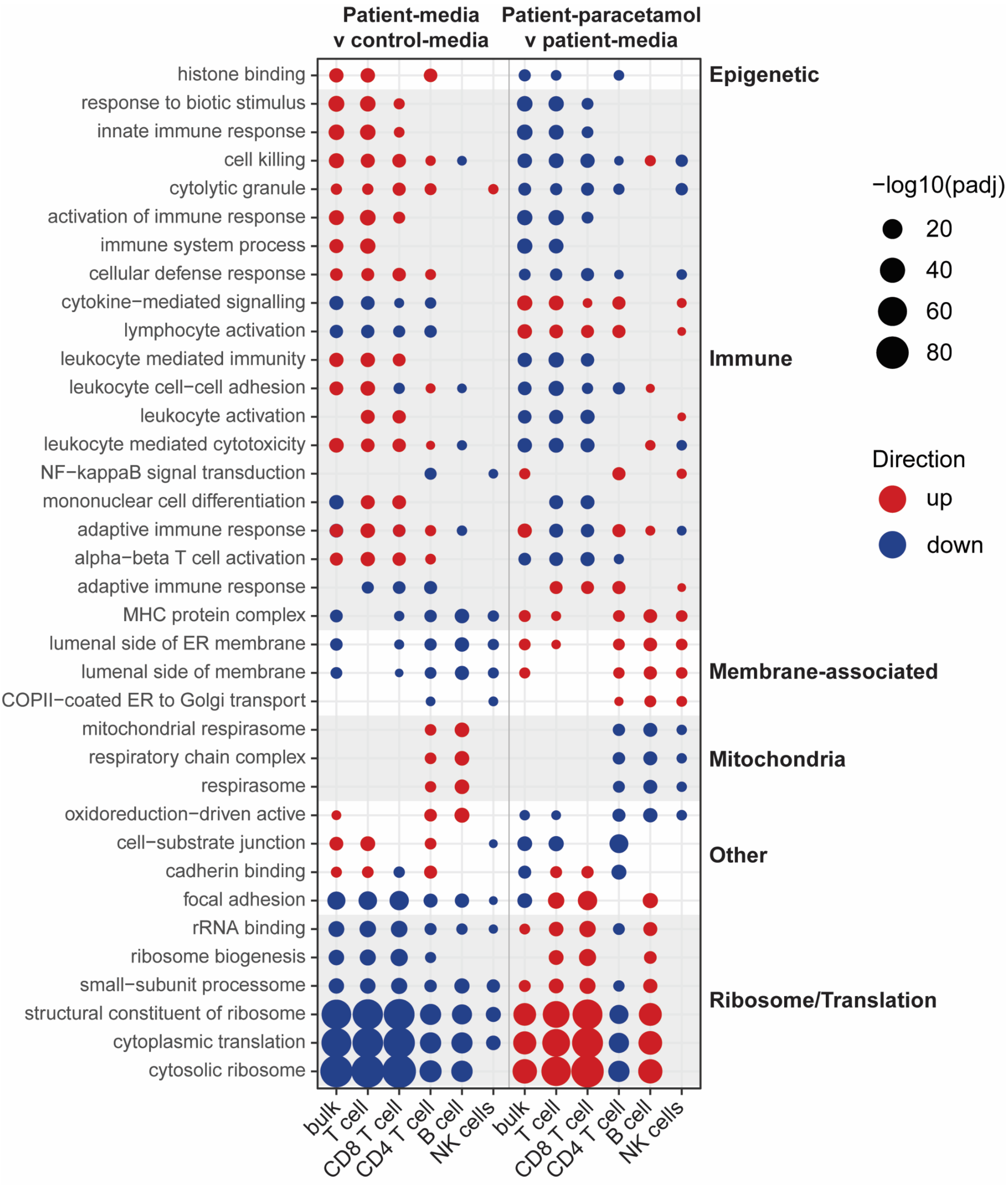

Top 5 upregulated (red) and downregulated (blue) pathways in bulk PBMCs, CD8 T cells, CD4 T cells, and B cells. Dot size represents –log10 FDR.

Supplementary Figure 4: Patient-ibuprofen versus patient-paracetamol dotplot

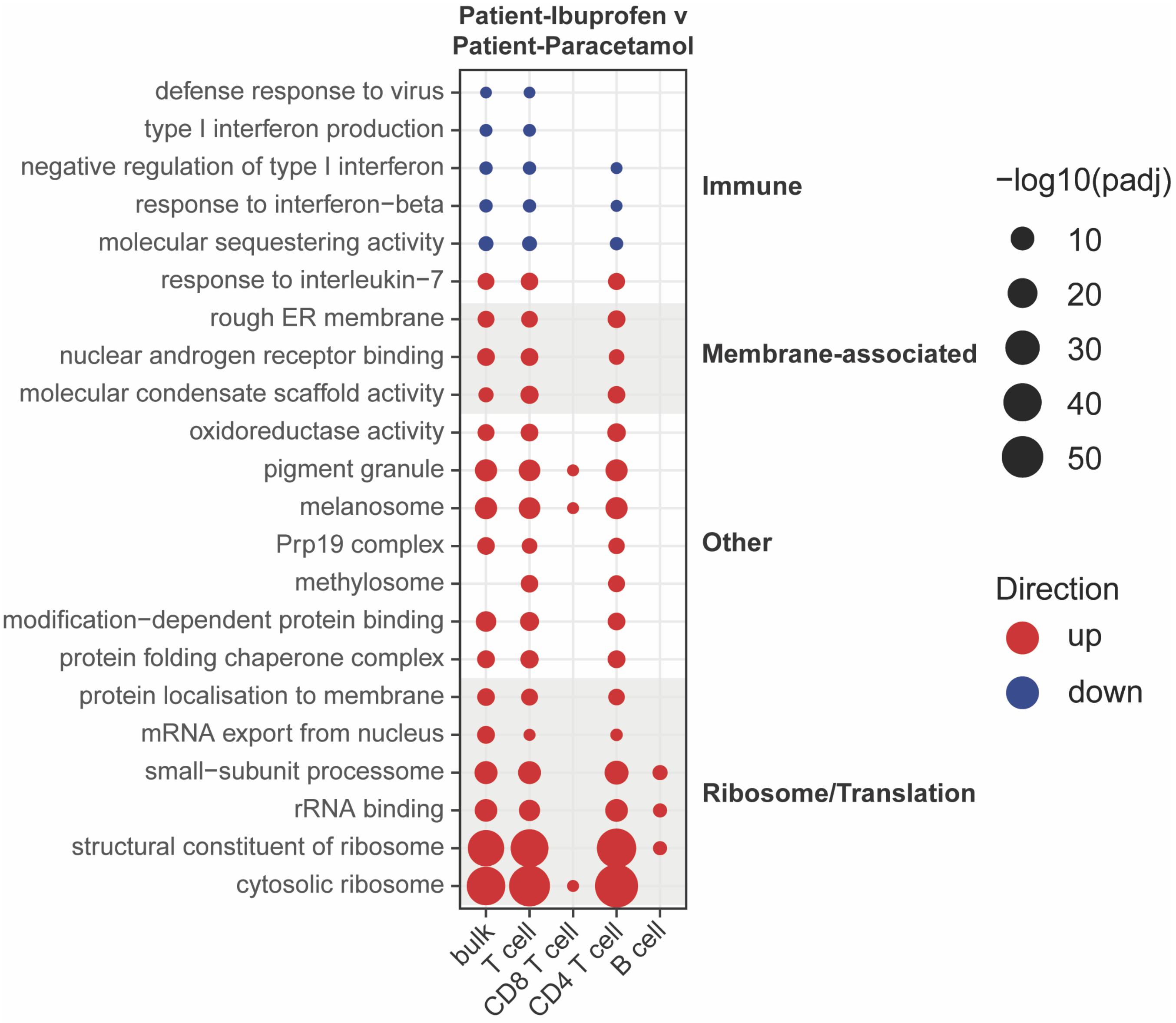

Top 5 upregulated (red) and downregulated (blue) pathways in bulk PBMCs, CD8 T cells, CD4 T cells, and B cells. Dot size represents –log10 FDR.

Supplementary Figure 5: Control-ibuprofen versus control-media dotplot

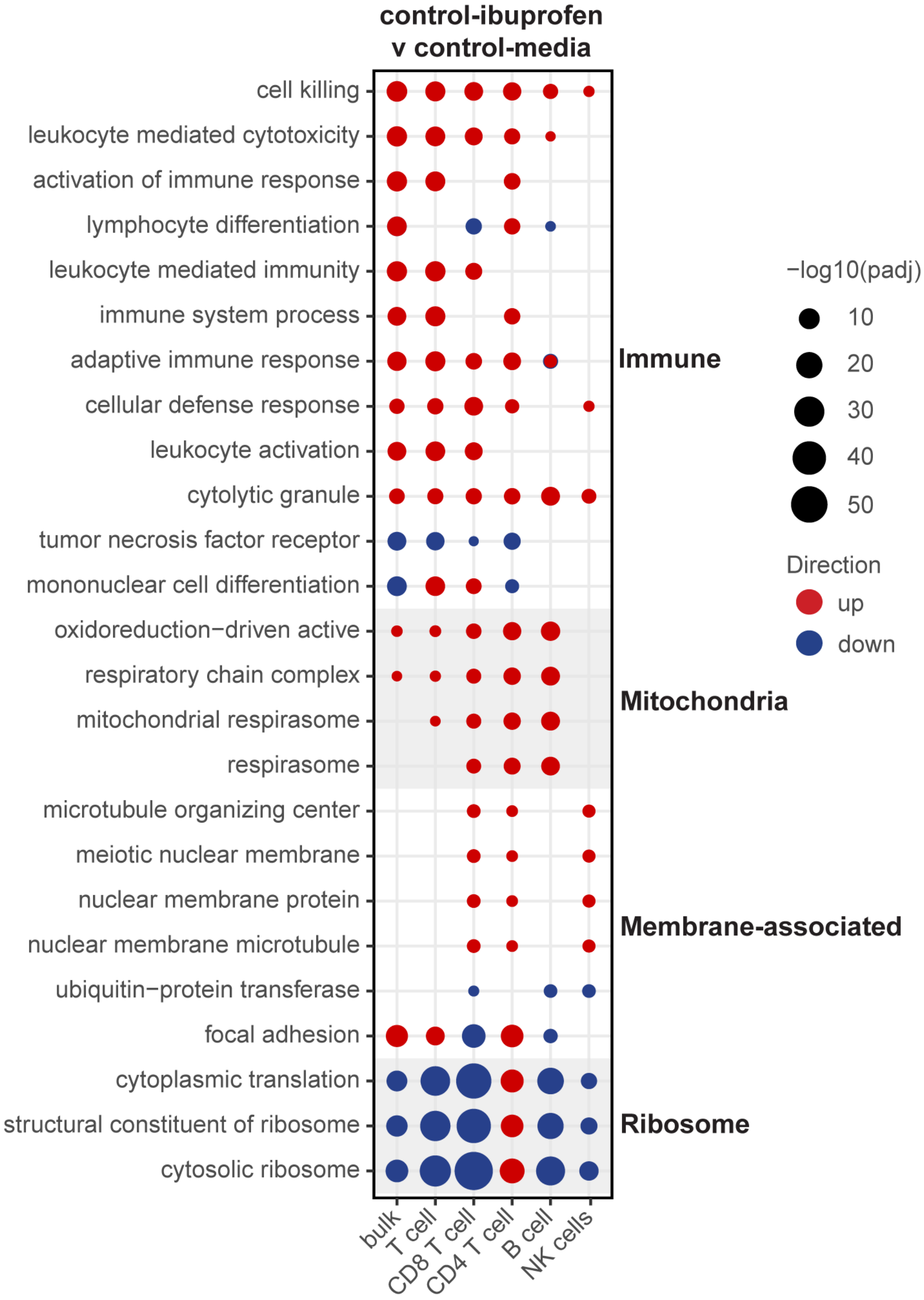

Top 5 upregulated (red) and downregulated (blue) pathways in bulk PBMCs, CD8 T cells, CD4 T cells, and B cells. Dot size represents –log10 FDR.
