# Supplementary material for: In vitro ibuprofen has gene regulatory and anti-inflammatory properties in peripheral blood mononuclear cells of individuals with infection-provoked neurodevelopmental disorders
Source: Inflammopharmacology. 2026 Jun 11;34(7):4951–62. doi: 10.1007/s10787-026-02282-7 (PMC13391669; doi:10.1007/s10787-026-02282-7)
Supplement: Supplementary file 2 [file 10787_2026_2282_MOESM2_ESM.docx]

Supplementary Methods

**Single-cell RNA seq workflow**

HIVEs^TM^ were then placed onto a spin plate and spun at 30 x *g* for 3 minutes to allow the single-cells to settle into picowells containing 3’ transcript-capture beads. The solution was removed from the HIVE^TM^ devices, and 2 mL sample wash solution (provided in kit) was added to each. The sample wash solution was removed, and the cell-loaded HIVEs™ were frozen at -80 °C after the addition of 2 mL cell preservation solution (provided in kit), before being transferred to Australian Genome Research Facility (AGRF Ltd, Westmead) and processed through to single-cell NGS libraries.

Following standard protocol, cell-loaded HIVE^TM^ devices were sealed with a semi- permeable membrane, allowing for the use of the strong lysis solution followed by the addition of hybridization solution. After collection, beads with captured transcripts were extracted from the HIVE^TM^ device by centrifugation. The remaining HIVE^TM^ library preparation steps were conducted in a 96-well plate format. The size distribution and quality of the final libraries were determined on a TapeStation 4200 platform with HD5000 ScreenTape System (Agilent Technologies, Santa Clara, CA, USA). The concentration of final pooled libraries was determined by qPCR. HIVE^TM^ scRNAseq libraries were sequenced using specific primers contained in the kit on an Illumina® NovaSeq® X platform (AGRF Ltd, Melbourne).

**Bioinformatic analysis**

Normalisation was performed using *SCTransform* and immune cell types were assigned with *scType* and *scPred*. Merged data were then split by cell type and normalised, scaled, and integrated between patients using harmony, then UMAP (uniform manifold approximation and projection) projections were made using the first 30 dimensions. Differentially expressed genes were identified using *FindMarkers.*
